# Electrochemical Oxidation of Ti-Grad 23 Alloy for Biomedical Applications: Influence of TiO_2_ Formation on Their Morphology, Composition, Wettability, and Chemical Corrosion

**DOI:** 10.3390/molecules31020251

**Published:** 2026-01-12

**Authors:** Lidia Benea, Nicoleta Bogatu, Veaceslav Neaga, Elena Roxana Axente

**Affiliations:** 1 Competences Centre—Interfaces-Tribocorrosion-Electrochemical Systems (CC-ITES), “Dunarea de Jos” University of Galati, 47 Domneasca Street, 800008 Galati, Romania; veaceslav.neaga@ugal.ro; 2 Interdisciplinary Research Centre in the Field of Eco-Nano Technology and Advance Materials CC-ITI, Faculty of Engineering “Dunarea de Jos” University of Galati, 47 Domneasca Street, 800008 Galati, Romania; 3 Center for Research and Technology Transfer in the Medico-Pharmaceutical Field, “Dunarea de Jos” University of Galati, 800008 Galati, Romania; elena.axente@ugal.ro; 4 Department of Pharmaceutical Sciences, Faculty of Medicine and Pharmacy, “Dunarea de Jos” University of Galati, 35, Al. I. Cuza Street, 800216 Galati, Romania

**Keywords:** electrochemical oxidation, Ti-Grad 23 alloy, surface morphology, phosphoric acid, X-ray diffraction, wettability, chemical corrosion

## Abstract

In this study, the influence of the electrochemical oxidation process on Ti-Grad 23 alloy (Ti6Al4V ELI) in 1 M H_3_PO_4_, under applied voltages between 200 and 275 V, at a constant time of 1 min, is analyzed. The structural, morphological, and wettability properties of the TiO_2_ anodic layers obtained were investigated by X-ray diffraction (XRD), energy dispersive electron microscopy (SEM-EDS), contact angle measurements, and chemical corrosion. XRD analysis showed the development and intensification of anatase and brookite phases, with increased crystallite size after electrochemical oxidation. SEM/EDS characterization confirmed the formation of an inhomogeneous porous TiO_2_ layer, with pore diameters ranging from 98 to 139 nm and a significant increase in oxygen content. Contact angle measurements demonstrate enhanced hydrophilicity for all oxidized samples, with progressively lower values as the applied voltage increased. Chemical corrosion tests in Ringer solution and Ringer + 40 g/L H_2_O_2_ indicated that oxidized surfaces maintain structural stability in physiological media, whereas exposure to oxidizing environments induces partial pore closure and crack formation due to localized corrosion. The optimal anodizing condition was identified at 200 V for 1 min, yielding a uniform distribution of pores and improved morpho-functional characteristics suitable for biomedical applications. The optimal electrochemical oxidation conditions were identified at 200 V for 1 min, ensuring a uniform pore distribution.

## 1. Introduction

Anodic oxidation is a well-established electrochemical process for the formation of oxide layers with insulating properties on metal surfaces, to increase their subsequent resistance to corrosion and wear in aggressive human environments [1,2,3,4].

The objective basis of electrochemical oxidation is to modify the surface properties of an alloy by creating an oxide layer in an aqueous electrolytic medium, using an external electric field as a driving force. Thus, the created coating can adopt either a solid form or a porous structure. However, the possibility of developing an amorphous or crystalline layer also depends on various factors, for example, the magnitude of the applied voltage, the duration of the process, and the type of post-treatment used [5,6,7].

Most studies in the field of electrochemical oxidation of metallic biomaterials are based on the exploitation of imposed conditions and parameters, such as the chemical composition of the electrolyte used, the temperature and duration of anodic oxidation, the magnitude of the applied voltage and/or the current density, but not least the type of substrate subjected to the process [4,8,9,10,11,12,13,14,15,16].

In the initial stages of development of electrochemical oxidation coatings, the essential elements, such as dilute electrolytes of sulfuric acid (H_2_SO_4_), acetic acid (C_2_H_4_O_2_), hydrochloric acid (HCl), or bases of sodium hydroxide (NaOH) and sodium sulfate (Na_2_SO_4_) as salt, favored the process of producing the protective TiO_2_ films [17].

Thus, Sul Young-Taeg et al. [18] in their experimental study demonstrated that dilute acidic or alkaline electrolytes can be used to create a compact nanoporous or microporous oxide layer. From a statistical point of view, this compact morphology of the oxide surface provides a complex and advanced interaction of the bone-implant contact [19].

Recent studies show that varying critical parameters in electrochemical oxidation can determine the surface morphologies, topographies, and biocompatibility classification of the anodic TiO_2_ layer. Thus, Long-Hao Li et al. [20] noted the need for a higher energy potential to disintegrate the dielectric (insulator) layer and, subsequently, to obtain a rougher surface with more hydrophilic properties of the oxide film on the surface of the titanium alloy. Fu-Yuan Teng et al. [21] studied the effect of voltages (100 V, 140 V, 180 V, and 200 V) applied in the electrochemical oxidation process of high-purity titanium samples in the electrolytic mix of H_2_SO_4_ and H_3_PO_4_. They discovered the advantage of the highest potential (in the experiment, 200 V) due to the increased roughness. The conclusion obtained is consistent with observations from other studies in the field [22,23,24,25,26,27].

Gelson B. de Souza et al. [28] using in their EC (electrochemical oxidation) study of commercially pure Ti by the galvanostatic method with a Ca-P-based electrolyte, observed in the layer obtained after the applied current density of 150 mA/cm^2^, the anatase crystallization phase (A) predominates with a higher hardness and modulus of elasticity compared to the surfaces of samples anodically oxidized at a density of 300 mA/cm^2^. However, the identification of the rutile phase (R), as well as the roughness, layer thickness, and pore size, increased concomitantly with the increased current density to 300 mA/cm^2^.

Carlos A.H. Laurindo et al. [29] studied the influence of high current densities (400, 700, 1000 and 1200 mA/cm^2^ for 15 s) in the Ca-P-based electrolyte environment, and found that when the current density exceeds a threshold of 1000 mA/cm^2^, the porosity and crystalline phases of rutile-type oxides (R) are considerably reduced, with the appearance of cracks in the thickness of the oxide layer. The predetermined time for electrochemical oxidation is another crucial aspect in modifying the surface properties of the anodic layer of Ti or Zr oxides. According to numerous studies, a longer anodization period results in a higher intensity of surface electric discharges, which results in the production of a larger and more crystalline anodic layer [30,31,32,33]. Usually, the formation of the nanotubular TiO_2_ oxide film is dependent on a longer anodization time at lower values of the applied potential. Where, for example, Baoe Li et al. [34] investigated the influence of the anodization period on the development of nanostructures in 1 M NaF medium at a potential (of 10, 20, 30 V) for 10 min, 30 min, 1 h and 4 h, and deduced that the surface roughness values of anodized Ti increase with increasing oxidation time, while the influence of voltage on the mentioned property is not significant.

However, among the conditions necessary to obtain the nanoporous oxide film, the high potential ratio at a short exposure time is observed, the values of these parameters vary, as in the case of an experiment that used a voltage of 70 V for a single minute to pass pure Ti in a 1M H_2_SO_4_ solution and obtained a three-dimensional network structure between the set of pores with the anatase phase of crystallization [35,36].

The electrolyte concentration in the electrochemical oxidation process is also an essential factor in determining the morphology or thickness of the oxide layer, thus the diameter of the nanostructures can vary, according to a research on the Ti6Al7Nb alloy in the case of using phosphoric acid solutions with different concentrations (1-3M H_3_PO_4_ with 0.4% HF by weight) [37], or using a solution containing 0.063 M CaF_2_ and 0.375 M NH_4_H_2_PO_4_ salts in the case of anodizing the Zr2.5Nb alloy [38].

Commercially pure titanium (CP-Ti) and the Ti6Al4V alloy are currently the most basic biomaterials used in the field of surgical implantology [39,40], due to their compositional variety and chemical characteristics such as excellent biocompatibility, good chemical stability in tissue environments and body fluids, or adequate mechanical properties, in particular the high Young’s modulus compared to human cortical bone [41,42].

The excellent corrosion resistance of Ti alloys is due to their affinity for oxygen, which, in turn, promotes the formation of an impermeable passive layer on their surface, largely composed of titanium oxides (TiO_2_, Ti_2_O_3_, and TiO) [43,44].

Due to its superior mechanical properties and corrosion resistance, Ti Grade 23 has been successfully promoted for use in orthopedic implants, such as those used for scapulohumeral and elbow joints, dental implants, tissue-engineered prostheses, cardiac pacemakers, intraocular lenses, and vascular stents. However, the tribological properties and fatigue behavior of this alloy require further improvements for use in highly loaded prostheses, such as hip and knee [45,46]. The bio-inert quality of the unprocessed titanium alloy on the contact surface does not provide complete osseointegration properties (with modest bone apposition) [47,48], with the provoking of local clinical pathologies (peri-implantitis, abscesses, etc.) [49,50], bacterial infections (with altered general conditions) [51,52], or with the subsequent need for systemic complementary therapies [53]. Another important factor is the exposure of the biomaterial to the aggressive biological environment of the body, which includes the interstitial fluid rich in electrolytes (Na^+^, K^+^, Cl^−^, PO_4_^−^) and plasma proteins. This interaction is abundant in the first stage of implant insertion or in the case of a local inflammatory process [54].

Currently, any in vitro or in vivo study of metallic biomaterials requires, as a first step, the creation of porous surfaces, which have a chemical composition and a crystalline structure appropriate to the environment in which they are to be implanted [55,56,57].

A properly developed coating should have mechanical qualities similar to the bone tissue of the patient undergoing surgery, if possible, a morphological imitation of bone structures.

The porosity of the obtained coating is essential in this context, since the size and shape of the pores directly influence the adhesion, proliferation, and differentiation of cells involved in tissue regeneration [38,58,59].

The crystalline phase of TiO_2_ significantly influences the biological response of titanium-based implants. In particular, the presence of the anatase phase has been associated with increased surface energy and improved hydrophilicity, favoring the adsorption of osteogenic proteins and promoting osteoblast adhesion, proliferation, and differentiation [60,61]. At the same time, the brookite phase, which is less thermodynamically stable, confers increased chemical reactivity to the surface, being reported in the literature as having a beneficial effect on reducing bacterial adhesion, by modifying the surface energy and local generation of reactive oxygen species [62].

In addition to porosity and crystalline phase, surface hydrophilicity also plays an important role in modulating the biological response of titanium implants. TiO_2_ surfaces with increased hydrophilicity facilitate rapid and stable adsorption of proteins from biological fluids, such as fibronectin and vitronectin, maintaining them in a conformation favorable for interaction with cellular receptors [60,63]. This process determines the efficient formation of cell adhesion complexes, leading to improved attachment, increased proliferation, and accelerated osteogenic differentiation of osteoblasts [60,63].

In addition, more hydrophilic surfaces can reduce initial bacterial adhesion by limiting hydrophobic interactions between the bacterial cell wall and the implant material, thus contributing to a decrease in the risk of microbial colonization [64].

According to Jung Park et al. [65], electrochemical oxidation is an effective method for controlling the geometry of the coating, aiming to increase the contact area between the bone and the implant or to generate a uniform bioactive layer of TiO_2_ nanotubes with a nanometric topography and appropriate roughness. This approach promotes implant osseointegration and prevents the subsequent formation of autoimmune reactive fibrous tissue, thus ensuring long-term orthopedic stability.

According to Raghuvir Singh et al. [66], corrosion resistance is an essential criterion in the selection of metallic biomaterials. The body fluids contained in the body (water, Cl^−^ ions, Na^+^ ions, proteins, and amino acids) create a corrosive environment that favors the release of toxic metal ions from alloys, which can compromise the stability of the prosthesis and cause systemic infections (in the liver, kidneys, heart, and skin) [67,68,69].

Benea L. and Celis J.P. [70] demonstrated that by modifying the surface of Ti6Al4V alloy using electrochemical oxidation in H_2_SO_4_ and coating with a chitosan layer, the corrosion resistance can be significantly improved. Tests performed in artificial Hank solution, which simulates blood plasma, showed that the combination of a porous TiO_2_ layer with chitosan reduces chemical reactivity and offers a promising concept for functional biomaterials. Anusha Thampi V.V. and collaborators [71] evaluated the anticorrosion properties of titanium surfaces modified by thermal spraying with Ti and Ta particles, followed by electrochemical anodization. Experiments showed that these treatments improve corrosion resistance in artificial solutions (PBS, SBF, and NaCl 0.9%), although, for anodized Ti/Ti-Ta samples in NaCl 0.9%, a lower resistance was observed [71].

The novelty of this study lies in the comprehensive evaluation of Ti-Grad 23 alloy anodized exclusively in 1 M H_3_PO_4_ under high voltages (200–275 V) for short exposure times, correlating for the first time the crystallographic evolution of TiO_2_ phases with pore formation, wettability enhancement and chemical corrosion behavior during 49 days in two different solution, to identify optimal oxidation conditions relevant for biomedical implant applications.

The study aims to identify the optimal anodizing conditions that enable the formation of homogeneous oxide layers with uniformly distributed pores and pronounced hydrophilic character.

## 2. Results and Discussions

### 2.1. Electrocrystallization of Ti-Grad 23 Alloy Evolution After Electrochemical Oxidation

**Figure 1:**
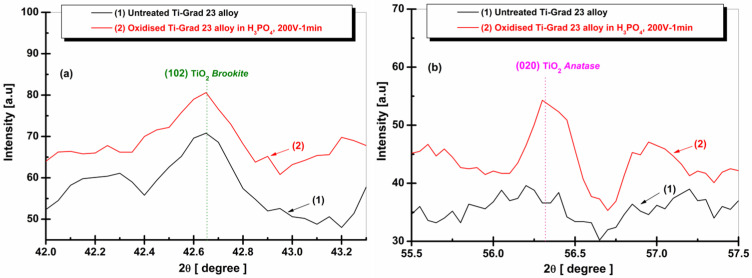
Comparative crystallographic planes of titanium dioxide obtained after electrochemical oxidation of Ti-Grad 23 alloy at 200 V for 1 min in H_3_PO_4_: (**a**) TiO_2_ Brookite (102); (**b**) TiO_2_ Anatase (020).

Figure 1 shows the evolution of the electrocrystallization of Ti-Grade 23 alloy after electrochemical oxidation at 200 V for 1 min in phosphoric acid in both crystallographic phases of TiO_2_ identified (Brookite (102) and Anatase (020)).

In Figure 1, the diffraction peaks corresponding to the Brookite (102) plane (Figure 1a) and the Anatase (020) plane (Figure 1b) show clear increases in intensity after electrochemical oxidation in H_3_PO_4_ compared to the untreated alloy. These increases indicate enhanced development and ordering of the crystalline TiO_2_ phases on the titanium surface, confirming that electrochemical oxidation in phosphoric acid promotes the formation of well-defined oxide structures.

**Figure 2:**
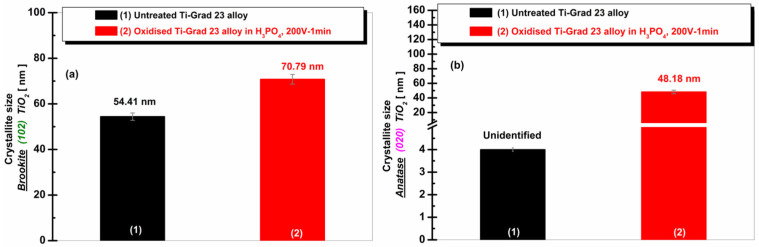
Average crystallite sizes developed on untreated Ti-Grad 23 alloy (1) and those oxidized at 200 V-1 min in H_3_PO_4_ (2) for each plane and type of titanium dioxide: (**a**) TiO_2_ Brookite; (**b**) TiO_2_ Anatase.

Figure 2 presents the average crystallite sizes formed on the surface of the Ti-Grade 23 alloy in the form of a column chart, allowing for a clearer visualization of the obtained values. The values were calculated using the Debye–Scherrer equation [72], shown in Equation (1).

(1) $$d_{XRD}=\frac{0.9\lambda }{FHWM\cdot \cos\theta }$$

According to this relationship, d_XRD_ is the crystallite size; the wavelength λ for Co K_α_ radiation is 1.790300 Å; FWHM (expressed in radians) represents the full width at half maximum intensity of the diffraction peak; and θ indicates the diffraction angle.

For the Ti-Grade 23 alloy, electrochemical oxidation in phosphoric acid leads to a noticeable increase in crystallite size for both TiO_2_ phases. In the case of the Brookite (102) phase, the crystallite size increases from the initial 54.41 ± 5.6 nm in the untreated alloy to 70.79 ± 4.2 nm after oxidation in H_3_PO_4_, indicating enhanced structural development of this phase. A similar trend is observed for the Anatase (020) phase, where the crystallite size reaches 48.18 ± 3.9 nm in the oxidized samples. Although the Anatase crystallites remain smaller than those of the Brookite phase, the overall increase in size confirms that oxidation in phosphoric acid promotes the growth and ordering of TiO_2_ crystallites on the alloy surface.

### 2.2. Contact Angle Evolution of Ti-Grad 23 Alloy Before and After Electrochemical Oxidation

Due to the wet biological environment, materials used in dental applications should have certain properties to avoid corrosion. The materials should also be hydrophilic for better biocompatibility performance. The adhesion properties of microorganisms on a biomaterial can be evaluated using contact angle measurements. Small water contact angles and high surface free energies indicate good adhesion properties of the material [73,74].

Contact angle studies provide information about the wetting properties of the implant material. Wettability may be one of the surface factors that should be considered when selecting biomaterials to be used for dental implants [74].

The average contact angle value obtained for untreated Ti-Grad 23 alloy and Ti-Grad 23 alloy electrochemically oxidized in phosphoric acid at 200 V, 250 V, and 275 V for 1 min is presented in Table 1.

The analysis of the data in Table 1 shows that the untreated alloy exhibits a contact angle of 87.08 ± 0.82°, which places the surface near the upper limit of the hydrophilic range. After oxidation in phosphoric acid, the contact angle decreases noticeably for all treated samples, reaching 69.67 ± 0.53° at 200 V, 65.93 ± 0.75° at 250 V, and 64.76 ± 0.38° at 275 V. In this context, the contact angle values obtained after electrochemical oxidation, which fall within the hydrophilic range, are consistent with surface characteristics reported in the literature to promote favorable protein adsorption, osteoblast adhesion, and a reduced tendency for bacterial attachment [60,63,64].

This progressive reduction in contact angle indicates a clear enhancement in surface hydrophilicity as the applied voltage increases. The improved wetting behavior can be attributed to the formation and growth of the oxidized TiO_2_ layer, which modifies the surface energy and promotes better interaction with the water droplet. Overall, the electrochemical oxidation process in H_3_PO_4_ leads to significantly more hydrophilic surfaces compared to the untreated alloy. Similar trends were observed in the literature by other authors [75].

### 2.3. Morphological and Compositional Analysis of Untreated Ti-Grad 23 Alloy Surfaces and Subsequently Obtained Oxide Films After Evaluation of Scanning Electron Microscopy (SEM-EDS) Data

**Figure 3:**
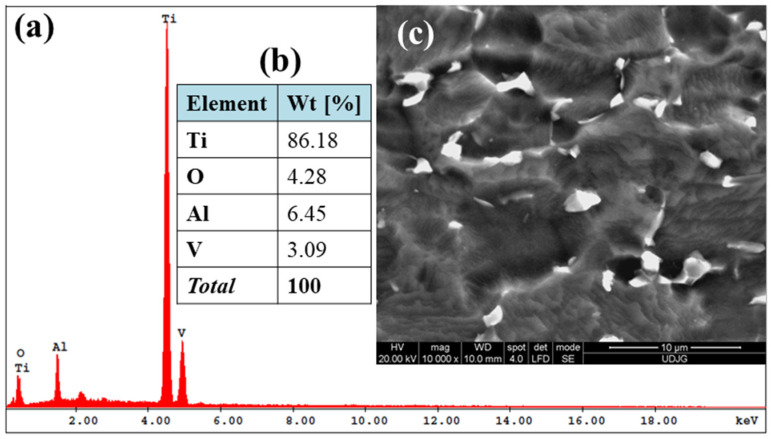
SEM-EDS data set of untreated Ti-Grad 23 alloy: (**a**) EDS spectral analysis; (**b**) EDS percentage analysis of the alloy compositional elements; (**c**) SEM micrograph.

Figure 3a–c illustrates the SEM-EDS data obtained as part of the initial analysis of the morpho-compositional characteristics of the Ti-Grad 23 alloy in its untreated state. The EDS analysis (Figure 3a,b) indicates the presence of the basic elements of the Ti-Grad 23 alloy. Thus, the mass percentage values of the untreated study samples are summarized as: Ti—86.18 ± 0.43%; Al—6.45 ± 0.26%; V—3.09 ± 0.21%. The amount of TiO_2_ for the untreated Ti Grade 23 alloy was estimated from the oxygen mass percentage measured by EDS, using the molecular weight of TiO_2_ (79.866 g/mol). Therefore, the TiO_2_ incorporated on the surface of the untreated titanium alloy, due to the native layer formed after exposure to the atmosphere following the manufacturer’s processing, is approximately 10.68 ± 0.23%.

It should be noted that in all EDS spectra performed, the presence of the gold peak was identified in the 2.12 keV range, because all treated and untreated study samples were gold-plated in an argon environment after an induced vacuum of 10^−1^ Pa for 10 s, to strengthen the surface electrical conductivity. Subsequently, the compositional calculation of the elements identified by the EDS spectra was reported with the exclusion of gold (Au) from the total amount.

From Figure 3c, it can be appreciated that the surface morphology at 10,000× magnification of the Ti-Grad 23 alloy is unevenly embossed, without mechanical post-processing defects, but with the visualization of oxides in the form of opaque and inhomogeneous structures.

**Figure 4:**
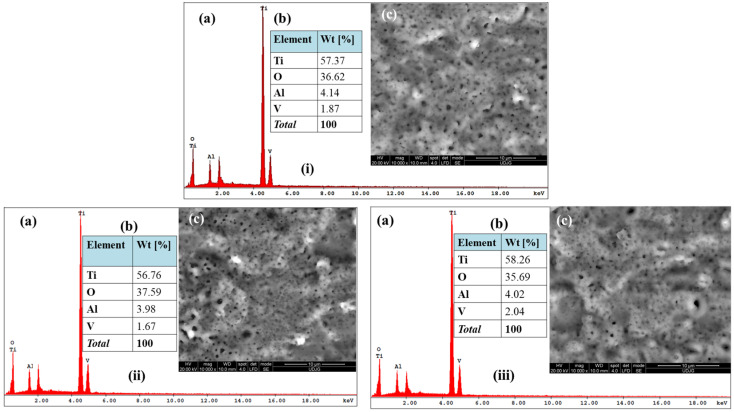
SEM-EDX data set of anodically oxidized Ti-Grad 23 alloy samples: (**i**) Ti-Grad 23 oxidized (200 V-1 min) in H_3_PO_4_; (**ii**) Ti-Grad 23 oxidized (250 V-1 min) in H_3_PO_4_; (**iii**) Ti-Grad 23 oxidized (275 V-1 min) in H_3_PO_4_: (**a**) EDS spectral analysis; (**b**) EDS percentage analysis of the oxidized alloy compositional elements; (**c**) SEM micrograph.

The compositional (EDX) and morphological (SEM) characteristics of Ti-Grad 23 alloy samples anodically oxidized in phosphoric acid (H_3_PO_4_) 1 mol/L are presented in Figure 4i–iii, where they are also compared after different applied potentials (200 V, 250 V, 275 V) for 1 min. Thus, it can be highlighted the significant increase in the mass percentage of oxygen for the samples oxidized in phosphoric acid compared to the untreated titanium alloy, where the estimated values in the case of titanium dioxide (TiO_2_) development on the modified surface are 91.45 ± 0.14% for 200 V, 93.77 ± 0.29% at 250 V and 89.10 ± 0.38% at 275 V applied potential, respectively. The differences in terms of the induced electrical potentials for oxygen growth are insignificant and do not present an evolutionary or involutive order, but a morphological structure with a lower number of defects can be noted through the presence of incompletely developed pores and with smaller variations in diameter, for the Ti-Grad 23 alloy anodically oxidized in 1M H_3_PO_4_ at 200 V for 1 min.

Similarly, it can be noted in the experimental study of Cheung K. H. et al. [76] that a mass percentage of oxygen of over 32.40% obtained after electrochemical oxidation of the Ti6Al4V alloy in 1M H_3_PO_4_ with an applied potential of 120 V for 10 min.

**Figure 5:**
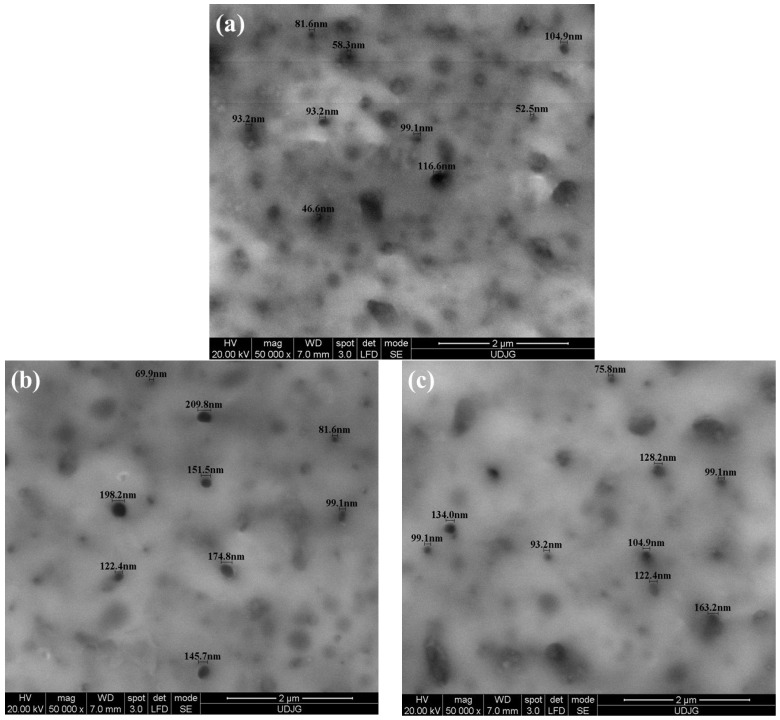
SEM micrographs at 50,000× magnification for electrochemically oxidized Ti-Grad 23 samples: (**a**) Ti-Grad 23 oxidized (200 V-1 min) in H_3_PO_4_; (**b**) Ti-Grad 23 oxidized (250 V-1 min) in H_3_PO_4_; (**c**) Ti-Grad 23 oxidized (275 V-1 min) in H_3_PO_4_.

The SEM micrographs in Figure 5 show the three groups of surface morphologies obtained at 50,000× magnification, samples of the Ti-Grad 23 alloy anodized in 1 mol/L phosphoric acid for 1 min at 200 V, 250 V, and 275 V (Figure 5a–c).

The SEM micrographs presented in Figure 5 reveal a distinct evolution of the morphology of the TiO_2_ layer formed by electrochemical oxidation as a function of the applied voltage. For the sample oxidized at 200 V for 1 min (Figure 5a), the surface exhibits a relatively uniform pore distribution with predominantly nanometric dimensions, the average pore diameter reported in Table 2 being mainly within the sub-100 nm range. The pores are well defined and homogeneously distributed across the surface, without the presence of extended regions with different morphological features. This morphology indicates a more controlled pore formation process compared to higher applied voltages.

For the sample oxidized at 250 V (Figure 5b), fully developed pores are observed; however, they display a larger size dispersion and a more heterogeneous surface distribution. At 275 V (Figure 5c), the pore boundaries become less clearly defined, and the surface shows increased heterogeneity, with regions where pores tend to merge or lose their individual morphological identity.

Similar SEM morphological results were also reported by Neide K. et al. [77], after anodic oxidation of commercial-pure Ti in 1.4M H_3_PO_4_ at 200 V and 250 V potentials for 1 min, revealed at 200 V structures with pores and craters formed on a relatively flat base oxide surface, and at 250 V potential the film morphology changes and presents smooth regions between the pores with predominantly round shapes. Table 2 presents the average values of the diameter of the nanopores obtained in the titanium oxide (TiO_2_) layer on the Ti-Grad 23 alloy electrochemically oxidized in 1M H_3_PO_4_ solutions.

The average values presented in Table 2 for the diameter of pores formed on electrochemically oxidized Ti-Grade 23 alloy samples indicate an evolution of their dimensions with increasing applied potential.

According to the classification criteria, pores with nanometric dimensions (below a tenth of a micrometer) were highlighted in the samples anodically oxidized at a potential of 200 V for 1 min, the average diameter being 98.10 ± 4.6 nm.

Another tool of the EDS spectral analyzer is the distribution maps of the identified elements. Therefore, in the case of Ti-Grad 23 alloy, the elements (O, Ti, Al, V) will be examined at a magnification resolution of 10,000× of the selected SEM images.

**Figure 6:**
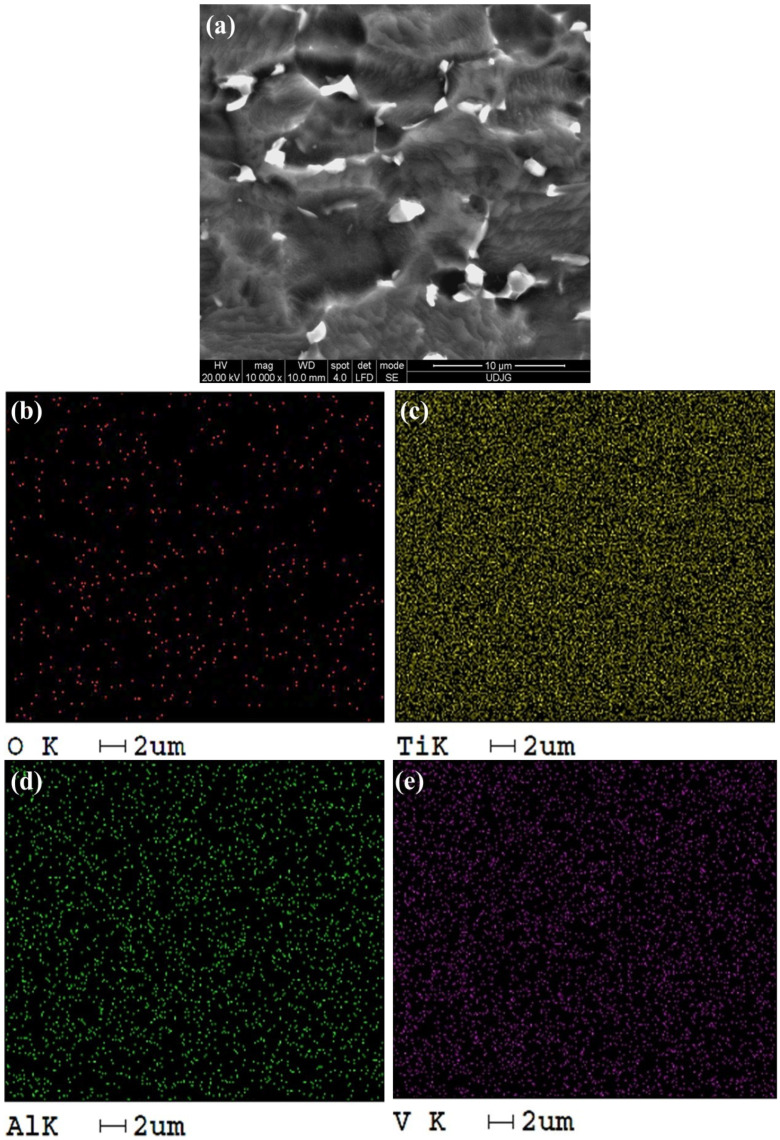
Distribution map of elements identified by EDS on the study surface of untreated Ti-Grad 23 alloy at a magnitude of 10,000×: (**a**) SEM image, (**b**) O element distribution, (**c**) Ti element distribution, (**d**) Al element distribution, (**e**) V element distribution.

Figure 6 shows the distribution maps of the basic elements delimited on the SEM-EDS study surface of the untreated Ti-Grad 23 alloy.

**Figure 7:**
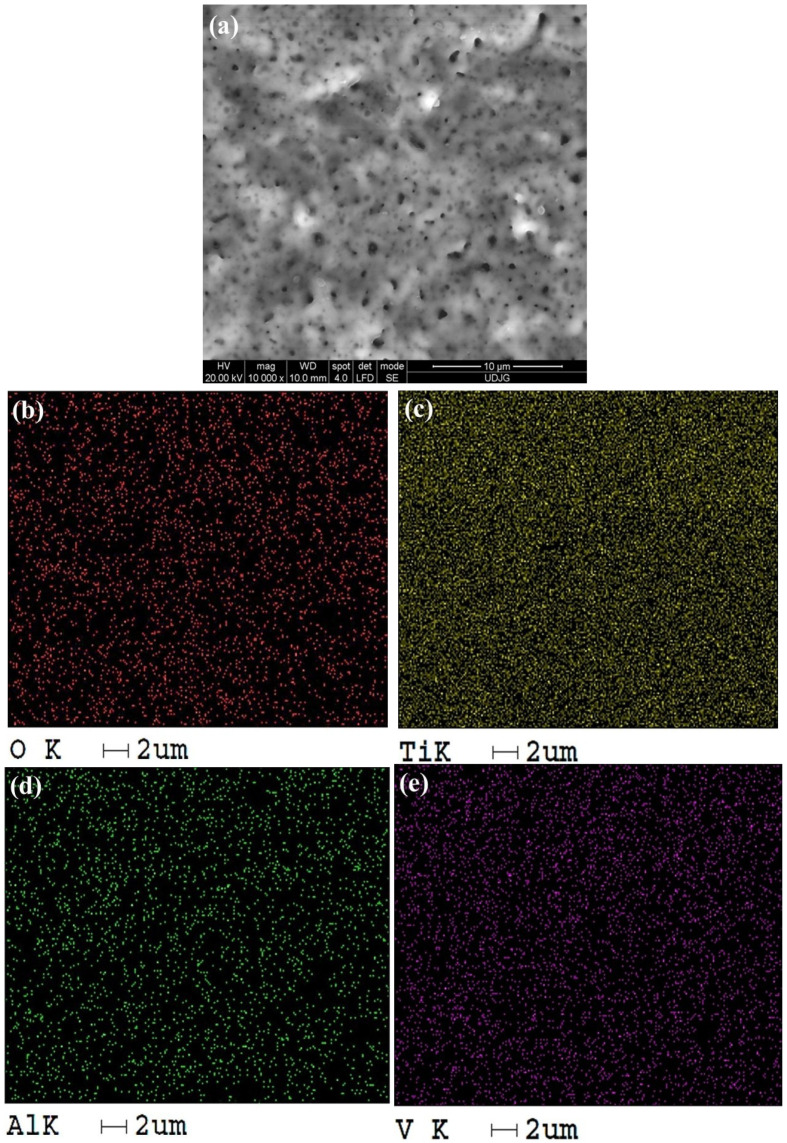
Distribution map of EDS elements identified on the study surface of Ti-Grad 23 alloy oxidized in 1M H_3_PO_4_ solution with a potential of 200 V for 1 min at a magnitude of 10,000×: (**a**) SEM image, (**b**) O element distribution, (**c**) Ti element distribution, (**d**) Al element distribution, (**e**) V element distribution.

In the case of Ti-Grad 23 alloy samples anodically oxidized in 1M H_3_PO_4_ at an electrical voltage of 200 V for 1 min, an increase in the oxygen distribution density can be observed compared to the untreated alloy, on the red pigmentation map in Figure 7b.

Similarly, a reduction in the distribution of the aluminum (Al) element can be observed according to the green pigmented map (Figure 7d, but this change is insignificant.

Analyzing Figure 6 and Figure 7, it was observed that the highest elemental distribution density is for titanium according to the yellow pigmented maps, thus in turn validating the mass fraction obtained after the EDS spectral analysis.

Similarly, the denser distribution of aluminum (Al) and vanadium (V) can be noted on the green and purple distribution maps, respectively (Figure 6 and Figure 7d,e), which disagrees with the mass fraction resulting from EDS analysis, due to the formation of aluminum and vanadium oxides concomitant with the increase in oxygen density on the examined surface.

Following the topography of the untreated Ti-Grad 23 alloy sample, an uneven relief without mechanical defects of finished processing can be highlighted, on the surface of which certain oxides with opaque white asymmetric structures are highlighted. After electrochemical oxidation of the Ti-Grad 23 alloy samples in phosphoric acid, an inhomogeneous film develops on these surfaces, comprising some alternating areas with small and large pores, in some places fully formed, and others without them as the voltage increases from 250 V to 275 V.

Comparative morphological analysis of the oxide layers formed in phosphoric acid as a function of the anodization potential indicates that the potential of 200 V is preferable to obtain the formation of well-defined mesoporous layers when electrochemical anodization is performed in phosphoric acid. This finding is in agreement with several scientific studies on the anodic oxidation of titanium alloys in phosphoric acid that report the formation of a corresponding porous layer for potentials from 150 V to 250 V [77,78,79,80,81].

### 2.4. Chemical Corrosion Assessment of Untreated Ti-Grad 23 Alloy Surfaces and Subsequently Obtained Oxide Films After Evaluation of Scanning Electron Microscopy (SEM-EDS) Data and XRD Analyses Before and After Immersion in Ringer and Ringer with 40 g/L H_2_O_2_

**Figure 8:**
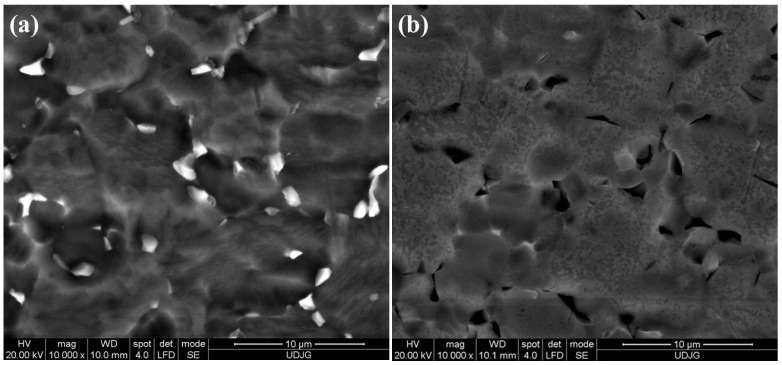
SEM morphology of untreated Ti-Grade 23 alloy sample after 49 days of immersion in (**a**) the Ringer solution and (**b**) Ringer solution with the addition of 40 g/L H_2_O_2_.

Figure 8a shows the surface morphology of the untreated Ti-Grade 23 alloy sample after 49 days of immersion in Ringer’s solution, while Figure 8b shows the corresponding morphology of the untreated Ti-Grade 23 alloy sample after 49 days of immersion in Ringer’s solution with the addition of 40 g/L H_2_O_2_.

By comparing Figure 3c before chemical corrosion with Figure 8a after chemical corrosion, it can be observed that after 49 days of immersion of the untreated alloy in Ringer’s solution, no evident morphological changes are present. In contrast, the untreated Ti-Grade 23 alloy sample immersed for 49 days in Ringer’s solution supplemented with 40 g/L H_2_O_2_ shows clear surface modifications, characterized by the formation of a more compact oxide layer.

The EDX values reported in Table 3 further support the SEM morphology described above. For the untreated Ti-Grade 23 alloy sample immersed in Ringer’s solution, the oxygen (O) content decreased by 1.78 ± 0.12 wt.% compared with the initial state (Figure 3b). A similar trend is also observed in the literature by other authors [82].

In the case of the Ringer + hydrogen peroxide solution, the compact oxide layer formed on the titanium alloy surface after immersion was confirmed by quantitative EDX analysis, which showed a substantial increase of approximately 6.62 ± 0.17 wt.% oxygen (O) relative to the initial values measured on the untreated control samples prior to chemical corrosion.

**Figure 9:**
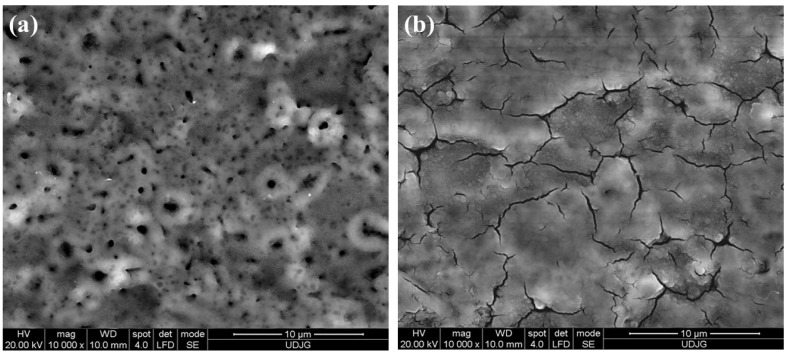
SEM morphology of Ti-Grad 23 alloy oxidized in 1M H_3_PO_4_ solution with a potential of 200 V for 1 min after 49 days of immersion in (**a**) the Ringer solution and (**b**) Ringer solution with the addition of 40 g/L H_2_O_2_.

Figure 9a shows the surface morphology of the Ti-Grad 23 alloy oxidized in 1M H_3_PO_4_ solution with a potential of 200 V for 1 min after 49 days of immersion in Ringer’s solution. Figure 9b shows the corresponding morphology of the Ti-Grad 23 alloy oxidized in 1M H_3_PO_4_ solution with a potential of 200 V for 1 min after 49 days of immersion in Ringer’s solution with the addition of 40 g/L H_2_O_2_.

If the Ti-Grade 23 alloy oxidized in 1 M H_3_PO_4_ with a potential of 200 V for 1 min before immersion (Figure 4i(c)) exhibits an oxide layer with a relatively homogeneous surface characterized by alternating regions containing a few well-defined pores and a greater number of incompletely formed pores that are predominantly distributed its morphology remains unchanged after immersion in Ringer’s artificial solution (Figure 9a), as evidenced by the SEM analysis.

However, for the samples immersed in the more aggressive medium composed of Ringer’s solution and hydrogen peroxide, the pores on the surface become obliterated, and the oxide layer appears opaque and partially compact due to the formation of numerous cracks, as shown in Figure 9b. These cracks form a reticulated network with irregular shapes and sizes, and their distribution is centered on the previously fully developed pores.

The formation of this structure can be attributed to localized corrosion of the sample surface induced by hydroxyl (OH^−^) radicals in the testing solution, a phenomenon also reported in other studies [83,84,85,86].

The EDX values recorded and presented in Table 4 further support the SEM morphology described above.

For the Ti-Grade 23 alloy oxidized in 1 M H_3_PO_4_ with a potential of 200 V for 1 min immersed in the Ringer solution, the oxygen (O) content decreased by 1.48 wt.% compared with the initial state (Figure 4i(b)).

In the case of the Ti-Grade 23 alloy oxidized in 1 M H_3_PO_4_ with a potential of 200 V for 1 min immersed in Ringer’s solution with hydrogen peroxide, the oxygen (O) weight percentage increased by approximately 1.62 ± 0.06 wt.% compared with the value recorded at the beginning of the test.

**Figure 10:**
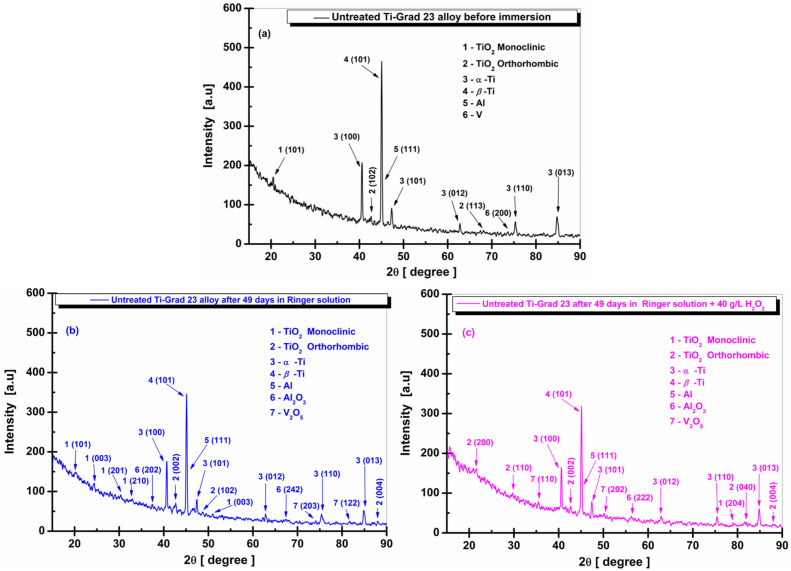
XRD spectra for untreated Ti-Grad 23 alloy: (**a**) before immersion; (**b**) after 49 days of immersion in the Ringer solution; and (**c**) after 49 days of immersion in the Ringer solution with the addition of 40 g/L H_2_O_2_.

In Figure 10a, the XRD spectrum for the untreated Ti-Grade 23 alloy before chemical immersion is presented. Index (1) identifies the crystalline phase of titanium dioxide (TiO_2_), which belongs to the monoclinic crystal system, space group P 1 21/m 1, according to the Crystallography Open Database (COD) 96-153-9683. This phase is detected only for the crystallographic plane (101) at the corresponding 2θ angle of 20.45°.

However, the distinctive crystalline phase of titanium dioxide (TiO_2_) of the brookite type, with an orthorhombic crystal system and space group Pbca, represented by index (2) in Figure 10a, was identified by the crystallographic planes (102) and (113) at the 2θ angles of 42.65° and 67.46°, respectively, corresponding to COD 96-900-4138.

In the case of titanium, the crystalline phases identified are divided into α-Ti, represented by index (3) in Figure 10a, with a hexagonal crystal system and space group P63/mmc, according to COD 96-901-6191. The crystallographic planes (100), (101), (012), (110), and (013) were observed at the corresponding 2θ angles of 40.56°, 47.26°, 62.74°, 75.27°, and 84.80°.

The other representative phase, indicated by index (4) in Figure 10a, is β-Ti, for which a single plane, (101), was identified at the 2θ angle of 44.98°, registered in the database under COD 96-900-8555, with a cubic crystal system and space group Im-3m.

Based on the XRD spectrum of the untreated Ti-Grade 23 alloy before chemical immersion, index (5) in Figure 10a corresponds to a crystalline phase of aluminum (Al). The crystallographic plane (111) was detected at the 2θ angle of 45.08°, with data correlated with the Crystallography Open Database (COD) 96-431-3211, where the crystal system is cubic, and the space group is Fm-3m.

The last phase identified, represented by index (6) in Figure 10a, is vanadium (V). According to the Crystallography Open Database (COD) 96-151-2550, vanadium has a cubic crystal system and space group Fm-3m, and only the crystallographic plane (200) was observed, corresponding to the 2θ angle of 73.65°.

The crystallographic planes for α-Ti (100, 101, 110) and β-Ti (101) have also been identified in other scientific studies on the Ti6Al4V ELI (Ti-Grade 23) alloy [87,88,89,90].

From the XRD spectrum of the untreated Ti-Grade 23 samples immersed in Ringer’s solution, Figure 10b, certain microstructural changes can be observed. These include the disappearance of the crystalline phase of vanadium (V) (COD 96-151-2550) associated with the crystallographic plane (200) corresponding to the 2θ angle of 73.65°, as well as the disappearance of the crystallographic plane (113) at 67.46° of orthorhombic titanium dioxide (TiO_2_).

At the same time, other products of chemical corrosion were identified, including the crystalline phase of titanium monoxide (TiO) according to the Crystallography Open Database (COD) 96-110-1050, with a monoclinic crystal system and space group A 1 1 2/m, identified by the crystallographic plane (131) at 50.80°. Titanium dioxide (TiO_2_), with a monoclinic crystal system and space group P 1 21/m 1, was also detected based on COD 96-153-9683 through the crystallographic plane (210) corresponding to 32.65°.

For TiO_2_ with an orthorhombic crystal system and space group Pbcn, the crystallographic plane (004) was identified at 87.14°, according to COD 96-900-4139.

In the case of aluminum oxide formed on the tested surface of the untreated titanium alloy, the phase Al_2_O_3_ was detected through the crystallographic plane (202) at 37.42°, as listed in COD 96-110-1169, where the crystal system is cubic, and the space group is Fd-3m.

The formation of vanadium pentoxide (V_2_O_5_) was also observed, through the crystallographic plane (203) at 74.16°, in agreement with COD 96-901-1016, corresponding to a monoclinic crystal system (space group C 1 2/m 1), and through the plane (010) at 24.21°, based on COD 96-101-1175, corresponding to an orthorhombic crystal system in the space group Pmn21.

Other crystalline phases identified exhibit no notable differences in peak intensity compared to the XRD data of untreated and non-immersed titanium samples.

The batch of untreated Ti-Grade 23 alloy samples statically immersed in Ringer’s solution with hydrogen peroxide, shown in Figure 10c, also displays several of the crystalline phases mentioned for the sample immersed only in Ringer’s solution, but with similarly low representative intensities and with different crystal systems.

Based on the XRD analysis, the crystalline phase of vanadium (V) (COD 96-151-2550) was absent, and the crystallographic plane (101) at 20.45° disappeared. In its place, another plane (204) was identified at 79.10°, corresponding to titanium dioxide (TiO_2_) with a monoclinic crystal system and space group P 1 21/m 1, according to COD 96-153-9683. The absence of the crystallographic plane (113) at 67.46° of titanium dioxide is also evident, accompanied by the identification of the crystallographic plane (040) at 82.02°, based on COD 96-900-4138, corresponding to brookite-type TiO_2_ with an orthorhombic crystal system and space group Pbca.

For titanium dioxide (TiO_2_) of the same orthorhombic type but belonging to the space group Pbcn, the crystallographic plane (110) was identified at 29.64°, according to COD 96-153-0027. It is noteworthy that a new crystalline phase of TiO_2_ with a triclinic crystal system and space group P1 was formed, in agreement with COD 96-412-4499, through the crystallographic plane (012) corresponding to 21.02°.

Aluminum oxide (Al_2_O_3_) was present only in a single crystallographic plane (222) at 56.35°, according to COD 96-100-0443, which represents an orthorhombic crystal system in the space group Pna21.

For the crystalline phase of vanadium pentoxide (V_2_O_5_), the crystallographic plane (110) at 29.68° was detected, consistent with COD 96-901-1016, corresponding to a monoclinic crystal system in the space group C 1 2/m 1.

Analysis of the X-ray diffraction patterns in Figure 10b,c shows that new oxide compositions (Ti–O, Al–O, V–O) were formed as a result of corrosion processes. Thus, for the untreated Ti-Grade 23 sample immersed in Ringer’s solution, a redistribution of oxygen content occurred in the newly formed O_2_-containing phases with a modestly distorted crystal lattice, compared with the alloy immersed in Ringer’s solution with hydrogen peroxide, where the crystalline structure exhibits more pronounced modifications. These compounds have also been reported in other experimental studies [91,92].

As a result of these effects, the diffraction reflection intensities of all titanium phases (α, β) present in the investigated composite decreased significantly.

The X-ray diffraction pattern of the untreated Ti-Grade 23 alloy immersed in Ringer’s solution (Figure 10b) reveals the appearance of peaks corresponding to Al_2_O_3_, V_2_O_5_, and TiO_2_ at lower diffraction angles, suggesting the presence of oxygen diffusion layers for all constituent elements.

The behavior of the untreated Ti-Grade 23 alloy immersed in Ringer’s solution with hydrogen peroxide is reflected by the development of a passive film primarily composed of TiO_2_, which is subsequently enriched with aluminum and vanadium oxides (Figure 10c).

By comparing the diffraction angles of the peaks corresponding to triclinic TiO_2_ with those of Al_2_O_3_ and V_2_O_5_, it becomes evident that aluminum and vanadium oxides appear at higher diffraction angles than titanium dioxide. One explanation for this is the higher solubility of aluminum and vanadium ions, which leads to the preferential dissolution of Al_2_O_3_ and V_2_O_5_ oxides.

Because the repassivation process occurs rapidly in an aggressive environment containing hydrogen peroxide, the Al_2_O_3_ and V_2_O_5_ particles occupy an increasingly smaller volume, and the surface passive film exhibits a non-uniform texture with equiaxed, non-dendritic solidification.

To effectively protect the underlying metal, the oxide layer must meet certain requirements, such as good adhesion to the metal, strong crack healing capability, high thermodynamic stability in the working environment, and low vapor pressure of the oxide [93].

**Figure 11:**
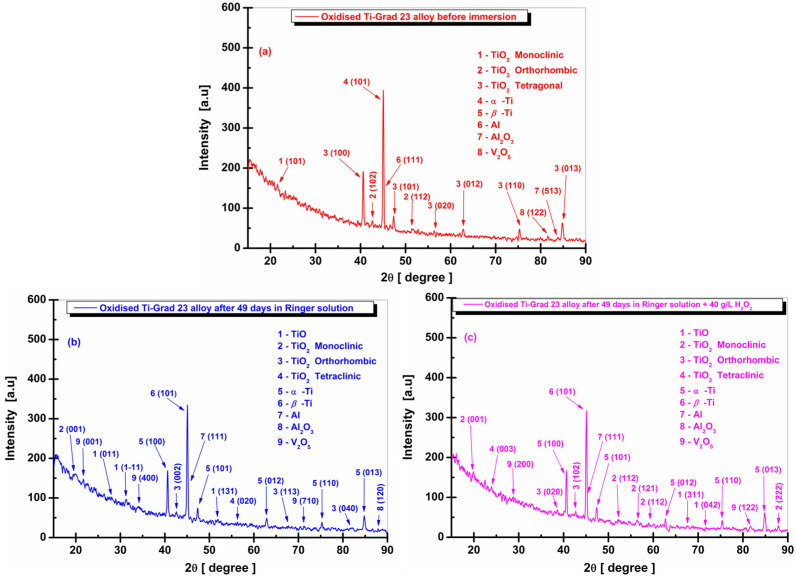
XRD spectra for oxidized Ti-Grad 23 alloy in 1 M H_3_PO_4_ with a potential of 200 V for 1 min: (**a**) before immersion; (**b**) after 49 days of immersion in the Ringer solution; and (**c**) after 49 days of immersion in the Ringer solution with the addition of 40 g/L H_2_O_2_.

In Figure 11a, the XRD spectrum of the Ti-Grade 23 alloy sample oxidized in 1 M H_3_PO_4_ at 200 V for 1 min before immersion is presented. By comparing Figure 10a with Figure 11a, it can be observed that, in addition to the phases identified in the electrochemically non-oxidized sample, a new crystalline phase of titanium dioxide (TiO_2_) of the anatase type appears. This phase is detected through a single crystallographic plane (020) at the corresponding 2θ angle of 56.37°, listed in the Crystallography Open Database (COD) 96-901-5930, with a tetragonal crystal system and space group I 41/amd.

Other observed modifications include the detection of additional crystallographic planes, such as (112) at the 2θ angle of 52.80°, corresponding to orthorhombic titanium dioxide (TiO_2_) with space group Pbcn, according to COD 96-153-0027.

Furthermore, aluminum oxide (Al_2_O_3_)— index (7) in Figure 11a—was identified with a cubic crystal system and space group Fd-3m through the crystallographic plane (513) at 83.75° based on COD 96-110-1169.

The last phase detected is vanadium pentoxide (V_2_O_5_), indicated by index (8) in Figure 11a. This compound crystallizes in an orthorhombic system with space group Pmn21, and only the crystallographic plane (122) was identified, corresponding to the 2θ angle 81.60°, according to COD 96-101-1292.

Thus, for the oxidized Ti-Grade 23 alloy samples immersed in Ringer’s solution, Figure 11b, the XRD analysis revealed the development of a crystalline titanium oxide (TiO) phase, identified according to the Crystallography Open Database (COD) 96-901-1016, with a monoclinic crystal system and space group A 1 1 2/m. The crystallographic planes (1–11) and (131) were detected at 2θ angles of 31.26° and 51.42°, respectively.

In the case of titanium dioxide (TiO_2_), also monoclinic, the crystallographic plane (101) at 20.45° reported in COD 96-153-9683 is replaced by plane (001) at 19.70°, identified according to COD 96-152-8779 with space group C 1 2/m 1. Orthorhombic TiO_2_ with space group Pbca, referenced from COD 96-900-4138, is represented only by the crystallographic plane (102) at 42.65°.

For tetragonal TiO_2_ with space group I 41/amd, listed under COD 96-901-5930, the crystallographic plane (020) at 56.37° is replaced by plane (116) at 82.25°.

Aluminum oxide (Al_2_O_3_) was identified in the XRD pattern by the crystallographic plane (022) at 32.84°, based on COD 96-100-0443, corresponding to an orthorhombic crystal system.

The other crystalline phase detected, vanadium pentoxide (V_2_O_5_), exhibited only one crystallographic plane, (001), at 21.86°, consistent with COD 96-901-1016 and characterized by a monoclinic crystal system.

For the electrochemically oxidized titanium alloy immersed only in Ringer’s solution, a slight reduction in oxide intensity was observed, but without structural changes in the porous layer formed after anodization.

In Figure 11c, the Ti-Grade 23 alloy oxidized electrochemically and immersed statically in Ringer’s solution with hydrogen peroxide, unlike the XRD results obtained in Ringer alone, shows no formation of the TiO crystalline phase.

At the same time, monoclinic TiO_2_ displays the space group P 1 21/c 1, in agreement with COD 96-901-5356, with two crystallographic planes detected: (112) and (121) at 2θ angles of 52.10° and 56.41°, respectively.

Monoclinic TiO_2_ with space group C 1 2/m 1, according to COD 96-152-8779, also appears, through plane (001) at 20.05°.

Based on the analyzed XRD spectrum, orthorhombic TiO_2_ (space group Pbca, COD 96-900-4138) is represented by a single crystallographic plane (020) at 38.75°. A newly identified differentiation is TiO_2_ of triclinic symmetry, space group P1, in agreement with COD 96-412-4499, where the crystallographic plane (003) appears with low intensity at 24.05°.

Additional changes observed in these samples include the aluminum oxide phase (Al_2_O_3_), where the crystallographic plane (111) at 22.40° corresponds to a cubic crystal system with space group Fd-3m, according to COD 96-110-1169.

Similarly, vanadium pentoxide (V_2_O_5_), identified through plane (122) at 81.55°, exhibits an orthorhombic crystal system (space group Pmn21), based on COD 96-101-1292.

In chemically aggressive conditions induced by hydrogen peroxide, oxidized Ti-Grade 23 alloy undergoes rapid diffusion of Ti ions to the alloy/solution interface due to their higher concentration and affinity within the alloy. Their interaction with oxygen ions leads to the formation of a TiO_2_ layer with a triclinic crystal system, detected at a very low diffraction angle, suggesting an increase in lattice parameters.

Following the formation of the Ti-rich oxide film, the underlying surface layers become depleted in titanium, and the aluminum concentration begins to increase in these regions [91]. As a result of outward diffusion of Al^3+^, cracks form on the outer surface of the oxide film, and their edges gradually accumulate Al_2_O_3_. Meanwhile, the vanadium oxide phase is reduced, as V-containing oxides cannot be detected at the outermost surface of the naturally formed oxide layers.

## 3. Materials and Methods

### 3.1. Materials

The titanium alloy Ti-Grad 23 alloy (Ti6Al4V ELI) used in this study is supplied by Goodfellow Cambridge, Huntingdon, UK, in the form of plates (300 mm × 300 mm × 1.5 mm) according to the ASTM B265 standard, and its mechanical properties and chemical composition are presented in Table 5 and Table 6.

After cutting the sheets of Ti-Grad 23 alloy at the dimensions of 2.5 mm × 2.5 mm × 1.5 mm, the samples are chemically degreased with a NaOH solution (50 g/L), then subjected to an attack with diluted hydrochloric acid (HCl 1:1) to remove organic impurities and superficial oxidations. Then, they are rinsed with distilled water to remove residues. To allow electrical connection, each sample is tied with a copper wire and covered with epoxy resin in order to have a 5 ± 0.3 cm^2^ active surface area.

### 3.2. Formation of Anodic Oxide Layers

**Figure 12:**
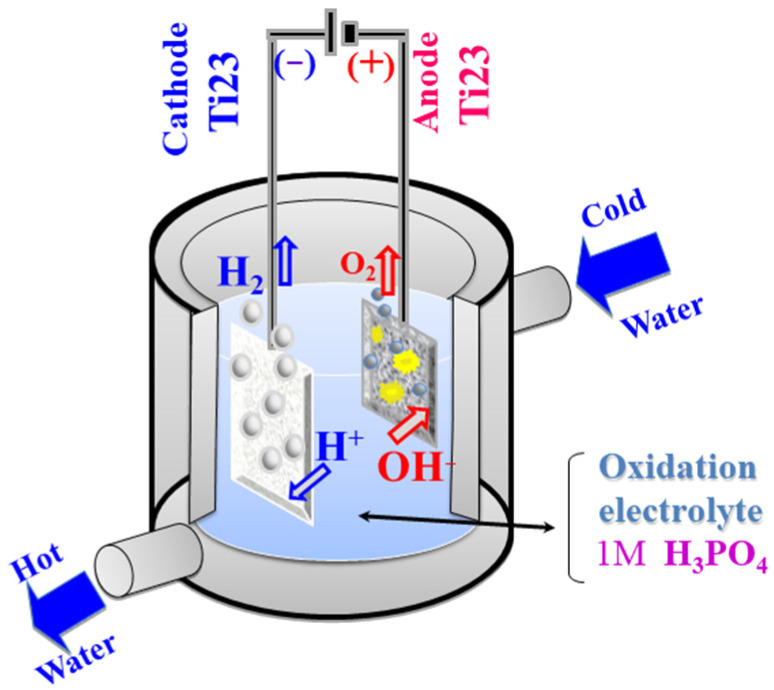
The electrochemical cell used for the oxidation of Ti-Grad 23 alloy.

The anodic oxidation was performed using a TDK Lambda GEN300-8 programmable power supply (TDK-Lambda Corporation, Tokyo, Japan) connected to a classical two-electrode electrochemical cell (Figure 12). The cell was equipped with an external cooling system to maintain a constant electrolyte temperature during the process. The electrolytes used were acidic solutions of 350 mL 1 M H_3_PO_4_.

The working electrode (anode—Ti-Grad 23 alloy) has an active surface area of 5 ± 0.3 cm^2^, and the cathode (Ti-Grad 23 alloy) has an area of 5 ± 0.2 cm^2^, a ratio chosen to ensure uniformity of the oxidation process. Before each experiment, the electrodes were prepared by immersion in ethanol and cleaning in an ultrasonic bath for 5 min, followed by rinsing with distilled water and air drying.

**Figure 13:**
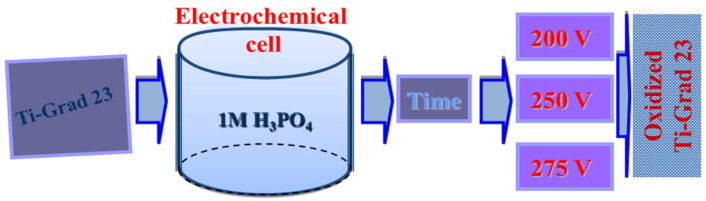
Schematic presentation of the experimental protocol of electrochemical oxidation applied to Ti-Grad 23 alloy samples.

The anodization processes were carried out at 22 ± 1 °C, in potentiostatic mode, by applying voltages ranging from 200–275 V and oxidation times of 1 min (Figure 13).

Although the anodization processes were investigated at different voltages (200–275 V) and different oxidation times, the results showed that the optimal parameters for obtaining pores with nanometric dimensions and uniform distribution were recorded when applying a voltage of 200 V for 1 min. All experiments were performed under identical conditions and were repeated five times to ensure the reproducibility and statistical validity of the data.

### 3.3. Mechanism of Anodic Oxidation Reactions

When the electrical circuit is closed, redox processes are initiated in the electrochemical cell: at the anode, oxidation of the substrate (Ti-Grad 23 alloy) occurs, releasing Ti^4+^ cations, while at the cathode, reduction reactions take place. The metal cations formed at the anode diffuse and react with oxygen from the water, causing the formation of a TiO_2_ layer on the alloy surface [1,95,96,97]. The reactions describing the main processes occurring at the interface are presented in Equations (2)–(5) [98,99]:

At the Ti/TiO_2_ interface:

(2) $$Ti\to Ti^{2+}+2e^{-}$$

At the TiO_2_/electrolyte interface:

(3) $$2H_{2}O\to 2{O_{2}}^{-}+4H^{+}$$

Oxygen ions react with titanium to form the following:

(4) $$2H_{2}O\to O_{2}(gas)+4H^{+}+4e^{-}$$

Some of the oxygen gas adheres to the electrode–electrolyte interface or is released into the atmosphere: Thus, at the end of the process, a compact layer of TiO_2_ is obtained on the anode surface.

(5) $$Ti^{2+}+2{O_{2}}^{-}\to TiO_{2}+2e^{-}$$

The formation of the oxide layer is governed by the migration of O^2−^ ions from the electrolyte to the metal/film interface and of Ti^4+^ ions from the metal substrate to the film/electrolyte interface [98]. The layer thickness increases almost linearly with the applied voltage, according to the relationship below (6):

(6) d=αU

where the following applies

d—thickness of the oxide layer,

α—growth constant (1–3 nm/V),

U—applied voltage [98,99].

The growth of the layer also depends on other factors, such as the distance between the electrodes, the oxidation time, the electrolyte temperature, and the applied current. However, this linear relationship is no longer valid once the layer reaches the critical thickness corresponding to the dielectric breakdown voltage of TiO_2_. Beyond this limit, the oxide film becomes unstable and loses its insulating properties [98].

### 3.4. SEM-EDS Characterization

The morphology and composition of the oxide films formed on Ti-Grad 23 alloy were investigated with a FEI QUANTA 200 scanning electron microscope (FEI Company, Hillsboro, OH, USA) equipped with an EDS analyzer (EDAX Genesis 5.10 software). To ensure the conductivity of the samples and prevent the accumulation of electrical charges, the surfaces were previously coated with a thin layer of gold (~10 s).

### 3.5. Structural Analysis (XRD)

Structural analysis was performed by X-ray diffraction using the Dron-3 diffractometer, with Co Kα radiation (λ = 1.790300 Å), at working parameters of 30 kV and 20 mA, in the range 2θ = 15–90°, with a step of 0.05°/s and a total acquisition time of approximately 2 h. The diffractograms were processed with Match! 3.16 software using the Crystallography Open Database (COD).

### 3.6. Contact Angle Analysis

The wettability properties were evaluated by measuring the static contact angle using a drop of Ringer solution (5 μL, 37 °C) deposited on the surface of the samples. The determinations were performed with the OCA 15EC goniometer (DataPhysics Instruments GmbH, Filderstadt, Germany) and SCA20 software version 4.3.19, applying the Young-Laplace method. Five measurements were performed for each sample, and the values were reported as arithmetic means.

### 3.7. Description of the Chemical Corrosion

The experimental study of in vitro chemical corrosion allows the investigation of the mechanisms of metallic ion release and the assessment of morphological changes occurring on biomaterial surfaces under conditions that simulate the internal environment of the human body. Through this method, the surface reactions occurring in an artificial physiological medium can determine the quantity and composition of the oxide layer, as well as the concentration of ions released into the solution [100].

The immersion tests followed the general methodology specified in ASTM G31-72 [101] using Ringer’s solution as the immersion medium, one of the most frequently employed artificial saline solutions in biomedical research [102,103,104].

The samples used in this study consisted of anodically oxidized Ti-Grade 23 alloy in phosphoric acid at 200 V for 1 min, as well as untreated (as-received) control samples.

These samples were subsequently subjected to morphological and structural evaluation after immersion.

The samples were immersed in glass containers filled with 150 mL of simplified Ringer’s solution and in the same physiological electrolyte supplemented with 40 g/L H_2_O_2_. The concentration of hydrogen peroxide was 30%. The tests were conducted for 49 days at 37 ± 1 °C. The chemical composition and physico-chemical parameters of simplified Ringer’s solution and simplified Ringer’s solution with 40 g/L H_2_O_2_ were described in another research paper [104].

During the immersion period, the solutions were gently agitated every 3 days to ensure medium homogeneity. At the end of the experimental period, the samples were thoroughly rinsed with distilled water and dried at room temperature. For reproducibility, three samples were tested in parallel for each study group. Under physiological conditions, the prevalence of bone tissue regeneration after implantation is approximately three months, which coincides with the remodeling phase of the native human tissue response [105]. This process may vary depending on the type of tissue involved, the age, the disease state, and whether the tissue defect is acute or chronic. When the defect becomes excessively inflamed, the process shifts toward chronic inflammation characterized by macrophage activity and lymphocytic infiltration, during which the local reactive compound hydrogen peroxide (H_2_O_2_) is continuously produced [106,107,108]. Certain biomaterials with immunomodulatory properties have the potential to enhance healing through their optimal morpho-structural characteristics, which support the adhesion of anti-inflammatory factors and the recruitment of specific immune cells [106,107,108].

The use of simplified Ringer’s solution supplemented with 40 g/L H_2_O_2_ generates a severe corrosion environment, significantly different from normal physiological conditions. This experimental choice was made deliberately, the solution being used as an accelerated corrosion environment, intended to simulate extreme oxidative conditions associated with local inflammatory processes, and not to accurately reproduce the in vivo environment.

The exact quantification of H_2_O_2_ concentrations generated in the peri-implant region is difficult to achieve, as local H_2_O_2_ levels can vary significantly depending on the intensity of the inflammatory response and the activity of immune cells [109,110,111,112,113,114,115]. For this reason, in vitro corrosion studies frequently employ elevated H_2_O_2_ concentrations to accelerate degradation mechanisms and to highlight relevant differences between surface treatments within a reasonable experimental period.

According to the scientific literature, the H_2_O_2_ concentrations used in simulated corrosion studies generally range between 33 mM and 300 mM, while some studies report much higher values [109,110,111,112,113,114,115]. Fei Yu et al. [110] investigated the effect of H_2_O_2_ concentration (0%, 0.1%, 1%, 10%, and 30%) on the corrosion resistance of the Ti6Al4V alloy, demonstrating that the addition of 10% hydrogen peroxide leads to an excessively aggressive environment, promoting the formation of Ti-H_2_O_2_ complexes and a quantitative increase in Ti ions in the test solution. At the same time, a concentration of 1% H_2_O_2_ can initiate oxidative processes at the level of the titanium alloy, without causing significant changes in the physicochemical parameters of the artificial solution [110].

The concentration of 40 g/L H_2_O_2_ (≈4% *w*/*v*) used in the present study was selected as a compromise between moderately oxidative conditions (<1%) and excessively aggressive environments (>10%), and is considered representative of a “worst-case” scenario.

This approach allows a comparative evaluation of the stability and effectiveness of anodic TiO_2_ layers under conditions of severe oxidative stress. At the same time, it should be emphasized that the results obtained from such accelerated tests cannot be directly extrapolated to the long-term behavior of implants in the real biological environment, but rather provide useful comparative information regarding the performance of modified surfaces.

In our experimental study, a 7-week immersion period was selected for both untreated and anodically oxidized Ti-Grade 23 alloy, representing an average duration also employed in other studies investigating the synthesis and collagen remodeling stages of biomaterials implanted in vivo [116,117,118,119,120].

## 4. Conclusions

The electrochemical oxidation of Ti-Grad 23 alloy in 1 M H_3_PO_4_ demonstrated a clear and consistent influence on the structural, morphological, and functional characteristics of the TiO_2_ layers formed under high applied voltages. The anodizing process promoted the development of anatase and brookite phases, accompanied by an increase in crystallite size, confirming the structural maturation of the oxide film. SEM–EDS analysis revealed the formation of a porous but heterogeneous TiO_2_ layer, in which regions with well-defined pores alternated with areas containing incompletely developed pores. The pore diameter increased progressively with applied potential.

Contact angle measurements showed a marked improvement in hydrophilic behavior after anodization, with the contact angle decreasing steadily as the voltage increased.

This enhanced surface affinity for aqueous environments supports the potential of oxidized Ti-Grad 23 alloy for biomedical applications.

Chemical corrosion tests confirmed the stability of the oxide layers in physiological environments. In comparison, immersion in the Ringer solution preserved the initial morphology, exposure to the oxidizing medium containing hydrogen peroxide induced partial pore closure and the appearance of surface cracks, without compromising the continuity of the oxide film.

Overall, the optimal oxidation condition is identified as 200 V applied for 1 min, leading to more uniform pore distribution and fewer structural defects. These studies demonstrate that anodization in phosphoric acid is an effective method for tailoring the surface of Ti-Grad 23 alloy, enabling the fabrication of TiO_2_ layers with properties suitable for integration into biomedical implants.

## Figures and Tables

**Table 1:** Droplet images and measured average contact angle values on untreated Ti-Grad 23 alloy and electrochemically oxidized Ti-Grad 23 alloy surfaces in 1M H_3_PO_4_.

Surface Studied	Contact Image of the Droplet with the Surface of the Untreated and Oxidized Alloy Study	The Average Contact Angle Values Obtained According to the Young–Laplace Method [Degree]
Ti Grade 23 alloy	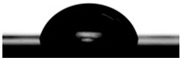	87.08 ± 0.82° Contact angle 0 < 90°, Hydrophilic surface
Ti-Grad 23_H_3_PO_4_ (200 V-1 min)	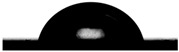	69.67 ± 0.53° Contact angle 0 < 90°, Hydrophilic surface
Ti-Grad 23_H_3_PO_4_ (250 V-1 min)	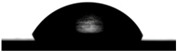	65.93 ± 0.75° Contact angle 0 < 90°, Hydrophilic surface
Ti-Grad 23_H_3_PO_4_ (275 V-1 min)	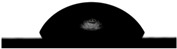	64.76 ± 0.38° Contact angle 0 < 90°, Hydrophilic surface

**Table 2:** Average values of the diameter of the nanopores obtained in the titanium oxide (TiO_2_) layer on the Ti-Grad 23 alloy electrochemically oxidized in 1M H_3_PO_4_ solution.

Surface Studied	Nanopore Diameter [nm]
Ti-Grad 23_H_3_PO_4_ (200 V-1 min)	98.10 ± 4.6
Ti-Grad 23_H_3_PO_4_ (250 V-1 min)	113.32 ± 10.9
Ti-Grad 23_H_3_PO_4_ (275 V-1 min)	139.20 ± 13.2

**Table 3:** Values identified after EDX analysis for untreated Ti-Grade 23 alloy samples after 49 days of immersion in the Ringer solution and Ringer solution with the addition of 40 g/L H_2_O_2_.

Chemical Element	Untreated Ti-Grade 23 Alloy Sample After 49 Days of Immersion in the Ringer Solution wt.%	Untreated Ti-Grade 23 Alloy Sample After 49 Days of Immersion in the Ringer Solution with the Addition of 40 g/L H_2_O_2_ wt.%
Ti	87.64 ± 0.91	80.95 ± 0.65
O	2.50 ± 0.15	10.90 ± 0.41
Al	6.51 ± 0.27	6.13 ± 0.11
V	3.35 ± 0.32	2.02 ± 0.09

**Table 4:** Values identified after EDX analysis for Ti-Grade 23 alloy samples oxidized in 1 M H_3_PO_4_ with a potential of 200 V for 1 min after 49 days of immersion in the Ringer solution and Ringer solution with the addition of 40 g/L H_2_O_2_.

Chemical Element	Ti-Grade 23 Alloy Oxidized in 1 M H_3_PO_4_ Sample After 49 Days of Immersion in the Ringer Solution wt.%	Ti-Grade 23 Alloy Oxidized in 1 M H_3_PO_4_ Sample After 49 Days of Immersion in the Ringer Solution with the Addition of 40 g/L H_2_O_2_ wt.%
Ti	58.85 ± 0.83	57.50 ± 0.55
O	35.14 ± 0.39	38.24 ± 0.24
Al	4.12 ± 0.16	3.26 ± 0.13
V	1.89 ± 0.07	0.99 ± 0.04

**Table 5:** Chemical composition of the purchased Ti-Grad 23 alloy [wt.%] [94].

C	H	Fe	O	N	Al	V	Ti	Others
0.03	0.003	0.1	0.13	0.01	5.5–6.5	3.5–4.5	Rest	0.3

**Table 6:** Mechanical properties of the purchased Ti-Grad 23 alloy.

Alloy	Modulus of Elasticity (Young) [GPa]	Breaking Strength [MPa]	Elongation δ [%]	Hardness HB [kgf/mm^2^]
Ti Grade 23	114	>860	15	33

## Data Availability

The original contributions presented in this study are included in the article. Further inquiries can be directed to the corresponding authors.
